# A contour property based approach to segment nuclei in cervical cytology images

**DOI:** 10.1186/s12880-020-00533-9

**Published:** 2021-01-28

**Authors:** Iram Tazim Hoque, Nabil Ibtehaz, Saumitra Chakravarty, M. Saifur Rahman, M. Sohel Rahman

**Affiliations:** 1 Department of CSE, BUET, ECE Building, West Palashi, Dhaka, Bangladesh; 2 Department of Pathology, Bangabandhu Sheikh Mujib Medical University, Shahabag, Dhaka, Bangladesh

**Keywords:** Cervical cancer, Image processing, Nuclei, Pattern recognition, Segmentation

## Abstract

**Background:**

Segmentation of nuclei in cervical cytology pap smear images is a crucial stage in automated cervical cancer screening. The task itself is challenging due to the presence of cervical cells with spurious edges, overlapping cells, neutrophils, and artifacts.

**Methods:**

After the initial preprocessing steps of adaptive thresholding, in our approach, the image passes through a convolution filter to filter out some noise. Then, contours from the resultant image are filtered by their distinctive contour properties followed by a nucleus size recovery procedure based on contour average intensity value.

**Results:**

We evaluate our method on a public (benchmark) dataset collected from ISBI and also a private real dataset. The results show that our algorithm outperforms other state-of-the-art methods in nucleus segmentation on the ISBI dataset with a precision of 0.978 and recall of 0.933. A promising precision of 0.770 and a formidable recall of 0.886 on the private real dataset indicate that our algorithm can effectively detect and segment nuclei on real cervical cytology images. Tuning various parameters, the precision could be increased to as high as 0.949 with an acceptable decrease of recall to 0.759. Our method also managed an Aggregated Jaccard Index of 0.681 outperforming other state-of-the-art methods on the real dataset.

**Conclusion:**

We have proposed a contour property-based approach for segmentation of nuclei. Our algorithm has several tunable parameters and is flexible enough to adapt to real practical scenarios and requirements.

## Background

Cervical cancer is the fourth most frequent cancer in women, affecting 490,000 new women each year, with more than 270,000 deaths [1]. With the introduction of simplified Papanicolaou (Pap) smear in 1957, Cervical cytology became the standard screening test for cervical cancer and premalignant cervical lesions. A *Pap test*, also called a *Pap smear*, is a medical examination that a doctor uses to detect potentially precancerous and cancerous processes in the cervix. Conventionally, the slides containing the Pap smear are examined under a microscope by a cytologist or pathologist. This manual procedure not only is very time and labor-intensive, but also is prone to error, intra-observer, and inter-observer variability. Such errors, which mainly concern false-negatives, used to be as high as 30% before the introduction of newer preparation techniques (e.g., SurePath [2], Thinprep [3]). These new preparation methods facilitate a reduction of the presence of cell clumps and elimination of blood, mucus and inflammatory cells in cytology specimens [4]. They also open up a whole new avenue in cervical cancer screening, namely, the automated screening with computer-based systems. However, the remaining presence of artifacts, superficial cells, and overlapping nuclei and cytoplasm remains as a critical obstacle for fully automatic cervical cancer screening.

The first stage of a computer-automated cervical cancer screening process is the segmentation of cervical cells. To do so, we first need to detect and identify the nucleus of a cervical cell, which is a segmentation task itself. Later stages use the detected nucleus to segment different overlapping and non-overlapping cell cytoplasm. Because each segmented nucleus indicates a cell, the result of this step (i.e., the efficacy thereof) directly affects the outcome of the final cytoplasm segmentation.

Techniques for nucleus segmentation in cervical cells have been well studied in the literature. An increasing number of studies have focused on this important problem owing to the event titled “Segmentation of Overlapping Cervical Cells from Extended Depth of Field Cytology Image Challenge” held during 2014 and 2015 under the auspices of the IEEE International Symposium on Biomedical Imaging (ISBI 2014, 2015) [5, 6]. The datasets for both challenges are publicly available, making the evaluation and comparison of different methods feasible. While a few of these methods are Machine Learning based, most have relied on Computer Vision techniques. Jung et al. [7] proposed a Bayesian Classifier based method. They used a distance transform and the EM (Expectation-Maximization) algorithm followed by Bayesian classification to segment overlapping nuclei. Keenan et al. [8] applied grey-level thresholding followed by contour following algorithms and morphological operations to segment and grade nuclei. Plissiti et al. [9] proposed a method based on a physical deformable model to do the same. Ushizima et al. [10] used a modified version of the local thresholding method proposed by Phansalkar et al. [11] to segment nuclei. Lu et al. [12] segmented nuclei by finding the Maximally Stable Extremal Regions (MSER) [13]. Lee et al. [14] segmented nuclei by performing local thresholding and by removing outliers based on features, such as, mean intensity, circularity, and size.

Saha et al. [15, 16] used fuzzy *c*-means clustering constrained by a Circular Shape Function (CSF) to detect a nucleus. Later on, Saha et al. [17] also proposed a method of segmenting cervical nuclei by merging the over-segmented SLIC superpixel regions based on pairwise regional contrast and image gradient contour evaluations. Their more recent work [18] proposes to segment nuclei by merging superpixels generated by the statistical region merging (SRM) algorithm using pairwise regional contrasts and gradient boundaries. Braz and Lotufo [19] used a deep learning convolutional network to detect and segment nuclei from pap smear images. Tareef et al. [20] introduced a novel method based on local distinctive features and guided shape deformation. In contrast, their more recent paper [21] introduced a multi-pass fast watershed-based method to segment nuclei and cytoplasms of a cervical cytology image. Although we focus in this paper only on cervical cytology images, we note that there exist notable researches on generic nuclei segmentation methods on various types of medical images (e.g., [22–24]).

All of the studies mentioned above on the segmentation of nuclei on cervical cytology images perform reasonably well on the ISBI datasets. However, since most of the studies were done based on the ISBI challenges, the performance of the respective methods on other independent (real) datasets remains unexplored. And our preliminary experiments suggested that several of the methods mentioned above, while excel in ISBI datasets, perform below par in another real dataset that we had collected. This motivated us to develop a more robust algorithmic pipeline that would work well on a dataset containing real cervical images in addition to the ISBI datasets.

In light of the above, we propose a novel approach for nucleus segmentation of cervical cells, which performs well on real-life cervical cytology images and improves the state of the art on the ISBI dataset. In particular, on ISBI datasets, our approach achieves a precision of 0.978 and recall of 0.933 with the F1-score being 0.955, the highest among all of the other state-of-the-art methods. On another dataset containing real cervical cytology images (BSMMU dataset), our algorithm achieves a promising precision of 0.770 and a formidable recall of 0.886 indicating that our algorithm can effectively detect and segment nuclei on real cervical cytology images. Moreover, through parameter tuning, we are able to increase the precision value to as high as 0.949 with an acceptable decrease of recall to 0.759. Our approach also managed a formidable Aggregate Jaccard Index of 0.681 outperforming other state-of-the-art methods in both pixel and object level performance measures. Thus our approach shows the promise to adapt to real practical scenarios and requirements.

## Methods

Every cervical cell contains a nucleus situated centrally within it. In many, if not most methods, segmentation of these nuclei is a prerequisite for cervical cell segmentation [4, 10, 12, 14]. The higher the accuracy of the nucleus segmentation, the better the cell segmentation will be as the presence of a nucleus confirms the existence of a cell around it. We describe our datasets, our nucleus segmentation algorithm, and the various tunable parameters thereof in the following subsections. We start with a brief description of some relevant notions and notations.

### Definitions

We define some terms here, most of which are related to the various contour properties we used in our algorithm. These definitions will facilitate a better understanding of our algorithm.

#### Contour size

Contour size is defined as the total number of pixels the contour is spanned throughout. It can also be defined in terms of the total area covered by the contour, but in our case, we have used the pixel count.

#### Solidity

Solidity is the ratio of contour area to its convex hull area as defined in the following equation.

1 $$\begin{aligned} Solidity = \frac{Contour~Area}{Convex~Hull~Area} \end{aligned}$$

#### Inertia ratio

Inertia Ratio is defined as the ratio of the length of the minor axis of an elliptical object to the length of the major axis.

2 $$\begin{aligned} Inertia~Ratio = \frac{Length~of~the~minor~axis}{Length~of~the~major~axis} \end{aligned}$$

### Datasets

We use two different datasets to train and evaluate our nucleus segmentation method respectively. The first dataset we use is publicly available (referred to as ISBI dataset henceforth) which was provided by ISBI in 2014 [5] containing 45 synthetic images for training along with 900 synthetic images and 16 real cervical cytology EDF (Extended Depth of Field) images for testing purposes. The synthetic images in the ISBI dataset were created by mirror transformations of background, and random rigid geometric and random linear brightness transforms of different annotated isolate cells in real EDF images [12]. The synthetic images have size $512 \times 512$, and each of them contains 2–10 different cells, while the real EDF images have size $1024 \times 1024$. All of the 45 training images and the 900 testing images are accompanied by their nuclei annotations for quantitative evaluation, whereas the EDF images are to be used for qualitative evaluation.

The second dataset we use is a private dataset (referred to as BSMMU dataset henceforth) collected from the Department of Pathology of Bangabandhu Sheikh Mujib Medical University (BSMMU), Dhaka, Bangladesh [25]. Ten cytology slides of cervical pap smear which did not have any diagnosed abnormality were randomly selected from the archive of the Department of Pathology, BSMMU. The slides were taken anonymously without any identifiable information about the patients, and therefore ethical approval was not required. These slides were prepared using BD SurePath$^{{\mathrm{TM}}}$ Liquid-Based Pap Test technology according to manufacturer’s instructions [26]. Papanicolaou staining procedure was used [27]. Each of the slides contained about 5000 epithelial cells, yielding approximately 50000 epithelial cells in total from all ten slides. All the slides were scanned using Hamamatsu NanoZoomer-SQ Digital Slide Scanner C13140-01 at the highest resolution (0.23 micrometer/pixel) under manual settings [28]. The images were saved in NDPI format with JPEG compression. We manually annotated 25 $250 \times 250$ sized real cervical cytology images among which 10 have been randomly selected to train the different tunable parameters of our algorithm while the rest 15 have been used for quantitative and qualitative evaluation. No validation set was used. The images contained 10–23 different cells along with their respective nuclei.

### Nucleus segmentation

In a cervical cytology image, nuclei are the most prominently visible regions. Commonly, they are relatively dark, uniformly shaped convex regions. Generally, they are circular or have an elliptical shape [4] except for some rare cases in the real cervical images, where, due to the 2D image being scanned from different depths, they may be somewhat irregularly shaped. We develop our algorithmic approach around four of the most visually distinctive properties of a nucleus: size, solidity, inertia ratio, and average intensity.

Before starting the actual nuclei segmentation procedure, a preprocessing step may be need to convert an RGB image into grayscale. The ISBI dataset is already in grayscale, so that doesn’t need further processing. The BSMMU dataset is in RGB. We have explored various ways to convert the images to grayscale. Firstly, we tried averaging the intensity values of 3 channels. Although this is the most rudimentary method of converting RGB to grayscale, it didn’t work well in keeping enough features for the nucleus segmentation to work properly. Secondly, we tried taking each channel separately. We also tried averaging two channels together, excluding the third one. Through careful observation, it became apparent that for the BSMMU dataset, the green channel of the RGB image contains the most amount of information, and so the best way to convert to grayscale is to take the green channel only. Thus, we converted the RGB images of the BSMMU dataset to grayscale by taking the green channel’s intensity value only. Notably, similar findings about the green channel have been reported in the literature as well (e.g., [29, 30]).

**Fig. 1:**
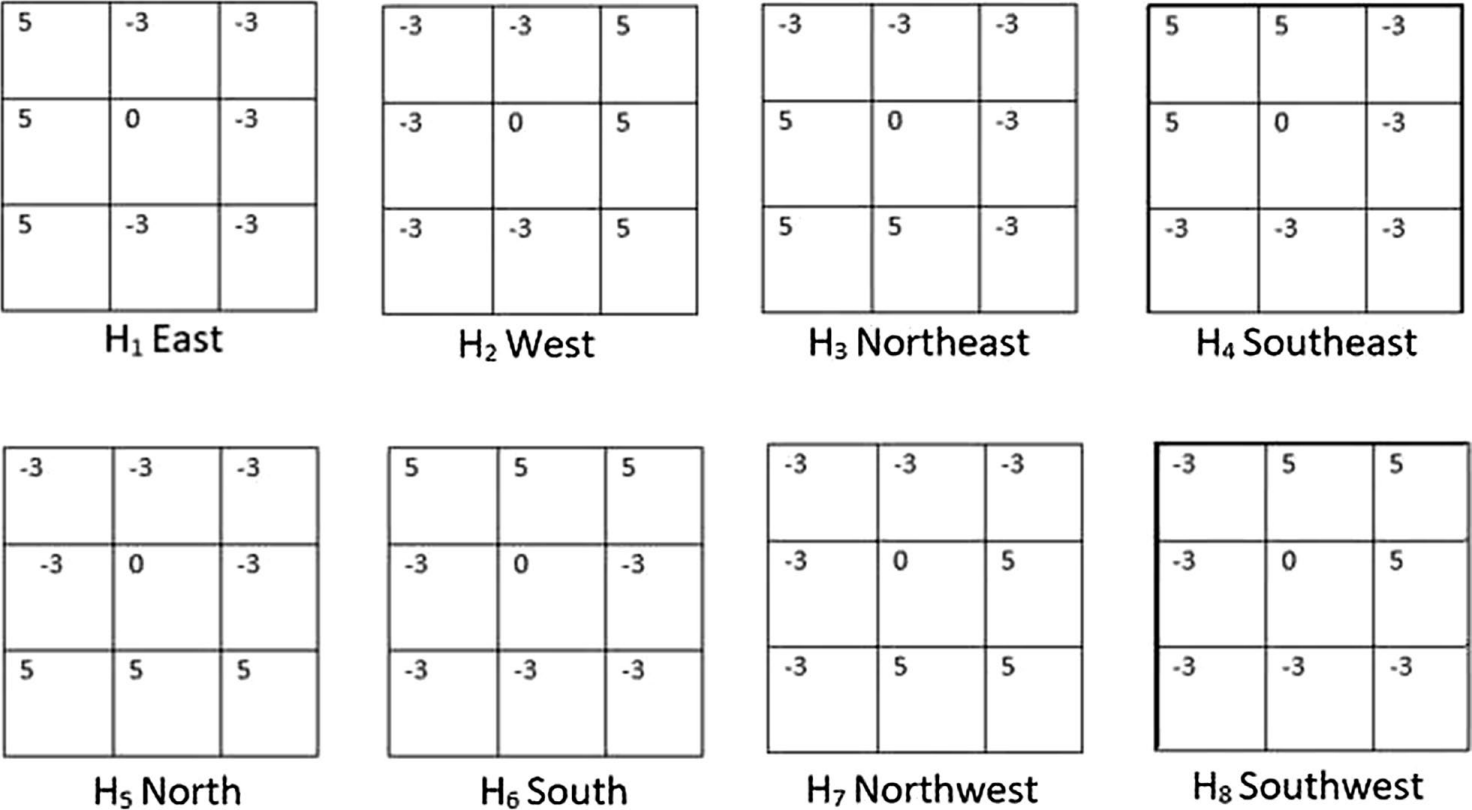
Impulse response arrays of Kirsch’s method

The cervical image is first smoothed using a Gaussian blur filter. Then adaptive thresholding is used since a nucleus is the darkest visible region within its cytoplasm. We use the built-in adaptive threshold function of OpenCV [31]. For this function, two parameters need to be carefully tuned, namely, the *window size* and the *constant “C”* (more details on these and other tunable parameters are presented in section "Tunable parameters"). The window size should be larger (smaller) for a dataset like the ISBI (BSMMU) dataset where nuclei are more zoomed in (out). The second parameter, i.e., the constant “C” gets subtracted from the mean or weighted mean calculated within the window. This constant needs to be smaller (larger) for an image where the contrast between the nucleus and the rest of the image is higher (lower). Due to overlapping cells, superficial noises, and artifacts, the thresholded image still contains various degrees of unwanted regions, more so in the real cervical images (i.e., BSMMU dataset). In the second stage, to reduce the number of unwanted regions, a convolution filter, which was implemented by Li and Chutatape [32] using Kirsch’s Method [33], is used. This filter computes the gradient of eight different directions by convolving the image with eight different template response arrays as shown in Fig. 1. The final gradient is set to the largest gradient. After that, a threshold is set to determine whether a pixel belongs to an edge or not. The final response contains various edges detected in the image [32]. Now, we do not actually need the edges detected here. But by subtracting this final response image from the previously global thresholded image, we can eliminate a large number of noises as follows. This filter’s response on the uniform, dense, and convex region is weaker than on the irregularly shaped non-convex regions. Thus, by subtracting this filter’s response from our thresholded image, we can remove many unwanted noises due to irregular shapes from the image. But this step has the undesirable side effect of reducing the size of the regions containing the actual nucleus, which we address in the later part of our algorithm.

In the next stage, we get all the contours detected from the thresholded image using the built-in contour detection function of OpenCV [31] and examine them one by one for contour properties. We calculate their size, solidity, and inertia ratio. Since the nuclei are uniformly shaped solid convex regions [4], they have pretty high solidity, always above 8.0 and most of the time above 9.0. So any contour with solidity lower than a preset minimum solidity value is rejected and removed from the image during this step. This minimum solidity is a tunable parameter as described in section "Tunable parameters". We also remove contours that are too small or too big in this step. The acceptable size can differ from dataset to dataset depending on the image’s zoom level and thus kept as a tunable parameter. We also reject regions with a low inertia ratio. Any region with a low inertia ratio is too elongated to be a proper nucleus; hence they are rejected. This minimum size, maximum size, and inertia ratio are also tunable parameters of the algorithm, which are described in section "Tunable parameters".

In the fourth and final stage, we recover the size of the nucleus regions, which were reduced during the second stage. This is an iterative procedure where the immediate neighborhood pixels of the nucleus region are checked one by one to see if they also belong in that region. The measure that determines the validity of the points is the intensity level. For a certain nucleus region, first, we compute the average intensity of all the pixels that already belong to that region. If a neighboring pixel of that nucleus region has an intensity value within a certain range of the average intensity of the nucleus region, then that pixel is deemed as a valid pixel, and subsequently, it is allowed to be part of that region thereby extending the nucleus region. This allowable average intensity range is also a tunable parameter of this algorithm (section "Tunable parameters"). During the parameter tuning stage (section "Tunable parameters") it was revealed that this range should be smaller (higher) for a dataset with low (high) contrast between a nucleus and outer cytoplasm. This iterative procedure continues until one of the three conditions is met:

1. No more valid pixel can be found from the immediate neighborhood of the contour boundaries.
2. The overall size of the contour (nucleus region) has become larger than the predetermined maximum size of a nucleus.
3. The solidity of the overall contour (nucleus region) has become smaller than a preset solidity value. This solidity value is set a bit lower than the usual solidity of a valid nucleus, which, from observation, is 0.8. A value of 0.75 works well here.

**Fig. 2:**
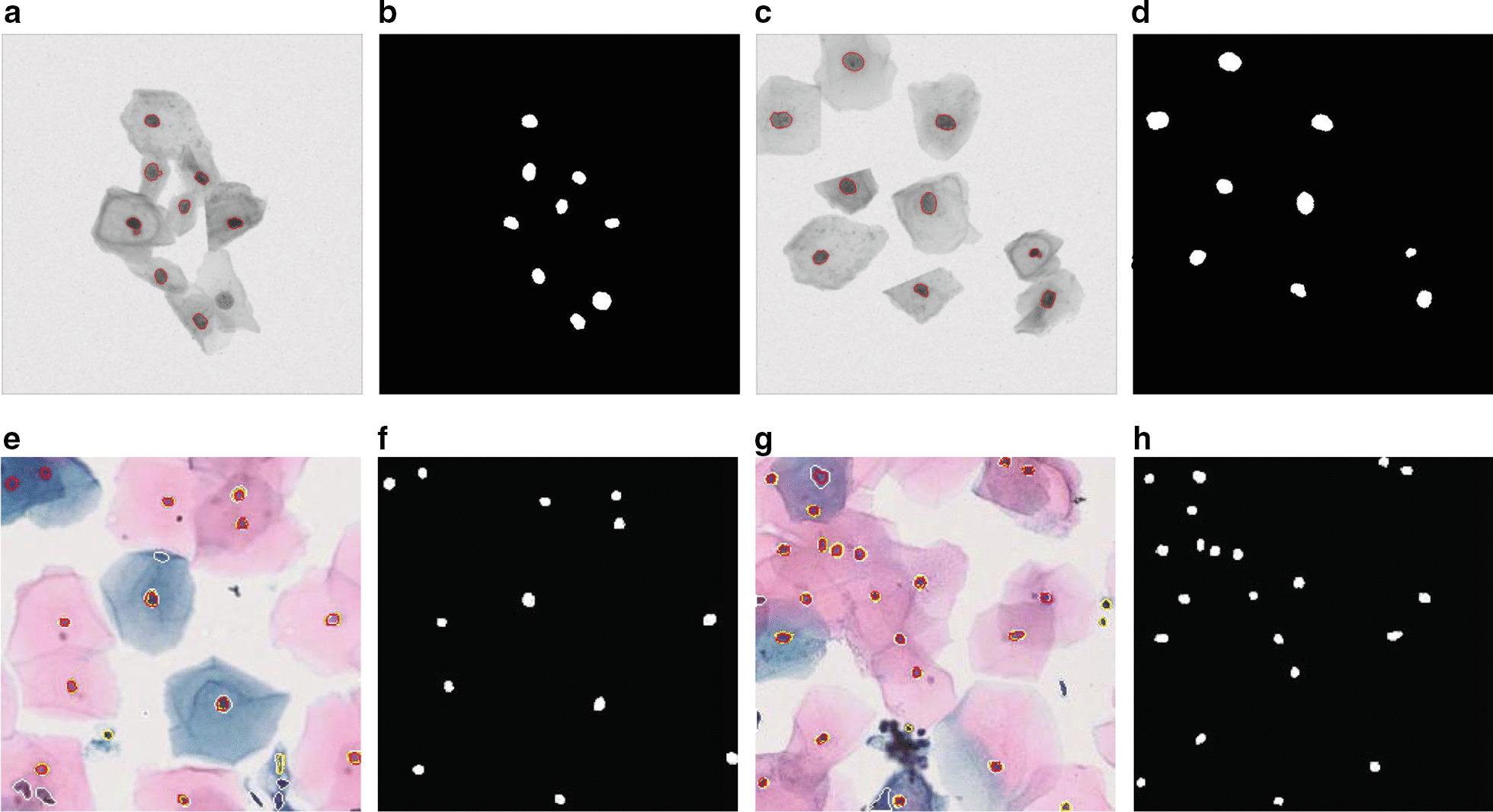
Nucleus segmentation on ISBI dataset (above: **a**–**e**) and BSMMU dataset(below: **e**–**h**) along with their corresponding Nuclei Annotations

Conditions 2 and 3 above act as checks against the uncontrollable growth of the regions in low contrast cervical cell images. Most cervical cell images have a high contrast between the nucleus and the cytoplasm and thus a carefully set acceptable average intensity range acts as the criteria to end this nucleus recovery procedure. But some cells have very low contrast. This can either be the trait of these cells due to high overlapping area, or it can be due to the image scanner focusing on the wrong depth when taking the cervical cytology image. The high or low contrast mentioned here doesn’t refer to any objective measure of contrast; rather it refers to subjective human observation. The parameter tuning stage of our approach, which is described in section "Tunable parameters", doesn’t need the objective measure of contrast. In any case, Conditions 2 and 3 essentially stop the overzealous growth of the regions. The steps of the algorithm are formally presented in Algorithm 1. Also, Fig. 2 shows our algorithm in action on some synthetic and real cervical cytology images. 
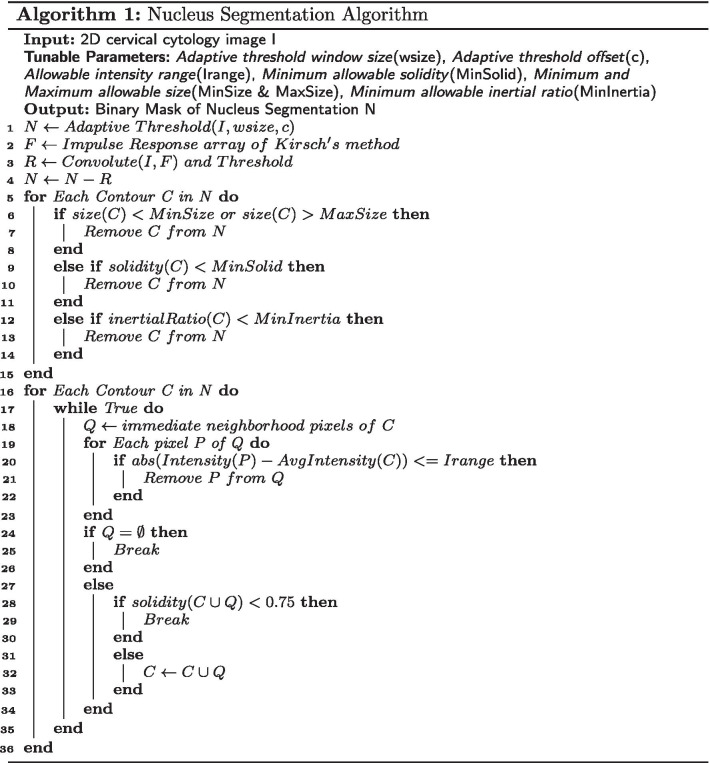

### Tunable Parameters

We have already briefly mentioned our tunable parameters while describing the algorithm in the previous section. In this section, we elaborate on those.

- *Adaptive threshold window size* (*wsize*): The window size is a tunable parameter because this needs to be larger (smaller) for the dataset where the nuclei are more zoomed in (out). Thus for the ISBI (BSMMU) dataset, *wsize* should be larger (smaller).
- *Adaptive threshold offset* (*C*): This offset value gets subtracted from the mean or the weighted mean calculated within the window. The value of *C* needs to be smaller for a dataset where the contrast between the nucleus and the rest of the image is higher and larger otherwise.
- *Range of intensity* (*Irange*): This value is defined as the intensity difference between the average intensity of the contour and the intensity of the neighborhood pixel being considered. The value of *Irange* should be smaller for a dataset where the contrast between the nucleus and outer cytoplasm is low and larger otherwise.
- *Minimum solidity* (*MinSolid*): This is the minimum solidity value which the contours in Stage 3 must conform with to be considered a valid nucleus. Usually, a value around 0.8 guarantees a very low amount of false positives while lower values can be used to allow more contours with the risk of higher false positives.
- *Minimum and maximum size* (*MinSize* and *MaxSize*): These values are used in Stage 3 to filter out too small or too big contours, which are considered noises. Their values depend on the zoom level of the nuclei in the dataset.
- *Minimum inertia ratio* (*MinInertia*): This is used in Stage 3 to filter out too elongated contours which are usually noises.

Our approach has seven tunable parameters. Manually tuning these parameters is undesirable and inefficient. This also makes the whole approach subjective to the dataset and hampers the generalizability. In order to circumvent this issue, we use a parameter tuning script on a small set (10 images are enough) of labeled training images to tune all of the parameters of our algorithm. This script runs a grid search on various combinations of all the parameters’ values and selects the combination that results in the highest value of the chosen performance measure. The script can be used to select precision, recall, F1-score, or Aggregate Jaccard Index (AJI) [22] as the performance measure to tune the values for the parameters of our algorithm. The procedure of calculating the different performance measures are described in section "Evaluation metrics". This parameter tuning script can be found in our online repository [34].

## Results

### Environments

We have conducted all our experiments using a single machine with Intel Core i3 CPU @ 3.6GHz (2 Cores, 4 Threads), 32 GB RAM, running Windows 10 Professional Edition (64-bit). We have used Python language (version 3.6.3 or above). The whole implementation was based on the OpenCV (version 3.4.7) library of Python. Helper libraries like Imutils (version 0.5.3) and Scikit-image (Skimage) (version 0.13.1) were used for utility functions to grab all contours from an image and get various contour properties like solidity, inertia ratio etc. from each contour. We also used the “signal” module of the Scipy (version 1.0) library for convolution of the images using 2D response filters during the noise removal stage.

### Evaluation metrics

To evaluate the effectiveness of our nucleus segmentation algorithm, we first use pixel-level *F-measure* or *Dice coefficient* to determine the validity of the detected regions. We then use the results to compute object-level performance measures, i.e., precision, recall and F1-score.

Dice coefficient is a pixel-level performance measure that measures the similarity between two regions. If a nucleus is represented by Region A in the ground truth and Region B in our nucleus segmentation mask, then Dice coefficient is defined as follows.

3 $$\begin{aligned} Dice = \frac{2 |A \cap B |}{|A |+ |B |} \end{aligned}$$

A Dice coefficient value of 0.6 means that there is 60% similarity between segmentation and ground truth. Dice coefficient has been extensively used in the literature to evaluate the performance of segmentation tasks (e.g., [4, 10, 12, 19]). Hence we have also used this measure to evaluate the validity of our nucleus segmentation as follows. If the Dice value for a nucleus is above or equal 0.6 then that nucleus is assumed to be correctly detected and is considered a True Positive (TP); otherwise, it is a False Positive (FP). If a nucleus in the ground truth is not even present in the nucleus segmentation mask, then it is considered a False Negative (FN). Finally, we compute the final performance measures, namely, precision, recall and F1-score (please see the equations below) thereby combining both pixel and object level performance measures in our experimental evaluation.

4 $$\begin{aligned} \mathrm {Precision}= & {} \frac{TP}{TP + FP} \end{aligned}$$

5 $$\begin{aligned} \mathrm {Recall}= & {} \frac{TP}{TP + FN} \end{aligned}$$

6 $$\begin{aligned} \mathrm {F1-score}= & {} \frac{2}{(Precision)^{-1} + (Recall)^{-1}} \end{aligned}$$

Although F1-score takes into account both precision and recall, we still separately report the latter two metrics because there can be various applications of nucleus segmentation and depending on the actual application setting, minimizing either FPs or FNs could be more desirable.

The combination of pixel-level Dice Coefficient and object-level Precision, Recall and F1-score is the most commonly used performance measure in the literature. However, according to [22], Aggregate Jaccard Index (AJI) encompasses both pixel level and object level performance in one measure. Therefore, we also included AJI in our comparative analysis on the BSMMU dataset.

### Results on ISBI dataset

Table 1 presents the results of our algorithm on the ISBI dataset in terms of Precision, Recall and F1-score and compares the results with that of the approach presented in [4] and two newer works in [15, 19]. As can be noticed from Table 1, our algorithm achieves the highest precision, highest recall (jointly with the work of Phoulady et al. [4]) and the highest F1-score. The values of the tunable parameters of our algorithm are reported in Table 2.

**Table 1:** Results on ISBI Dataset

Approaches/algorithms	Precision	Recall	F1-score
Ushizima et al. [10]	0.959	0.895	0.926
Lu et al. [12]	0.903	0.893	0.898
Lu et al. [36]	0.977	0.883	0.928
Phoulady et al. [37]	0.874	0.930	0.901
Saha et al. [15]	0.918	0.915	0.916
Braz and Lotufo [19]	0.929	0.917	0.923
Phoulady et al. [4]	0.961	**0.933**	0.947
**Our algorithm**	**0.978**	**0.933**	**0.955**

The best results are highlighted using boldface font

**Table 2:** Values of the Tunable parameters of our algorithm

Parameters	wsize	C	Irange	MinSolid	MinSize	MaxSize	MinInertia
ISBI	55	40	30	0.8	20	140	0.4
BSMMU (Case 1)	35	50	40	0.8	5	50	0.3
BSMMU (Case 2)	41	48	40	0.6	5	50	0.2
BSMMU (Case 3)	35	50	40	0.8	5	70	0.4

### Results on BSMMU dataset

To evaluate the performance of our algorithm and that of the algorithm of Phoulady et al. [4] on real cervical cytology images, we use the 15 annotated test images from the BSMMU dataset as described in section "Datasets". To run the algorithm of [4], we have used the implementation found in [35].

Table 4 reports the performance of both the algorithms on real cervical images in terms of precision, recall, F1-score, and Aggregate Jaccard Index (AJI) [22]. We report two separate cases of our algorithm with different values for the tunable parameters exhibiting the trade-off between the precision and recall. We also report another additional case showcasing the best-case scenario in terms of AJI [22]. The values of the parameters are reported in Table 2. In Case 1, our algorithm achieves very high precision at the cost of a lower recall (than that of Phoulady et al. [4]). For Case 2, our algorithm achieves high recall at the cost of somewhat lower precision. For Case 3, our algorithm achieves high AJI at the cost of a low recall value. Although not clearly mentioned in [4], through an in-depth review, we have identified some tunable parameters in the algorithm of Phoulady et al. [4]. These parameters are minimum nucleus size, minimum solidity, lowest threshold, highest threshold and minimum area and outer boundary average intensity difference. We also tune these parameters in an attempt to derive three results like ours, where the first one achieves high precision, the second one achieves high recall, and the final one achieves high AJI. For this algorithm, the best-case scenario for AJI is also the best-case scenario for precision value, thus case 1 and case 3 achieve identical results. Unfortunately, no combination of parameter values of their algorithm was able to achieve a decent precision. We still report all three cases. The values used for the parameters are reported in Table 3.

**Table 3:** Values of the Tunable parameters in the algorithm of Phoulady et al. [4]

Parameters	minsize	minsolid	lowthreshold	highthreshold	intensitydiff
ISBI	110	0.9	60	150	15
BSMMU (Case 1)	30	0.8	60	150	15
BSMMU (Case 2)	30	0.6	60	150	30
BSMMU (Case 3)	30	0.8	60	150	15

## Discussion

In this section, we briefly discuss some salient features of our approach.

### Our algorithm can balance precision and recall in real cervical cytology images

Our proposed method has achieved very high precision and recall on the (benchmark) ISBI dataset as reported in Table 1. In fact, it has achieved the highest precision and recall among all the previous works making it the current state of the art.

Subsequently, we put our algorithm to test by running it on the BSMMU dataset. While the results on the real cervical images (i.e., BSMMU dataset) are not as stellar, they are still very impressive. Even though our algorithm did not achieve high precision and high recall simultaneously (Table 4), it is flexible enough to achieve high precision or high recall while keeping the other decently high. This flexibility is undoubtedly desirable for practical purposes as discussed below.

**Table 4:** Result on BSMMU dataset

	Precision	Recall	F1-score	AJI
Ahmady Phoulady et al. (2017) (Case 1)	0.656	0.873	0.749	0.490
Ahmady Phoulady et al. (2017) (Case 2)	0.643	0.895	0.750	0.488
Ahmady Phoulady et al. (2017) (Case 3)	0.656	0.873	0.749	0.490
**Our algorithm (Case 1)**	**0.949**	**0.759**	**0.845**	**0.556**
**Our algorithm (Case 2)**	**0.770**	**0.886**	**0.822**	**0.489**
**Our algorithm (Case 3)**	**0.938**	**0.558**	**0.700**	**0.681**

Currently, a computer-based, fully automated diagnosis of the pap smear test is not available. We don’t actually foresee or even desire this to happen; we want to keep computer-based diagnosis systems as an assistant to the human medical practitioners. In this context, our algorithm is expected to be used as follows. In our scenario, only the output of our algorithm, i.e., the identified nuclei, are expected to be examined and analyzed by the pathologists. So, high recall is very important here since we don’t want to miss many nuclei. Precision is not as important in this case because the pathologist, while analyzing later, can attribute the false positives as noise himself (and discard those). Precision, while not as significant as recall in this scenario, is certainly not a throwaway measure since higher precision means less false positives, meaning less annoyance for the pathologist analyzing the pap smear for diagnosis.

Our work can be considered as the first step in the pap smear test pipeline. Machine learning based algorithms can be applied on cells defined by the identified nuclei, and automated diagnosis could be carried out to aid the pathologists further. In such a case (or in the case of a fully automated cervical cancer cell segmentation method), precision will become a highly significant measure as false positives, in that case, may result in false diagnoses.

### Our algorithm delineates nuclei boundaries better

**Fig. 3:**
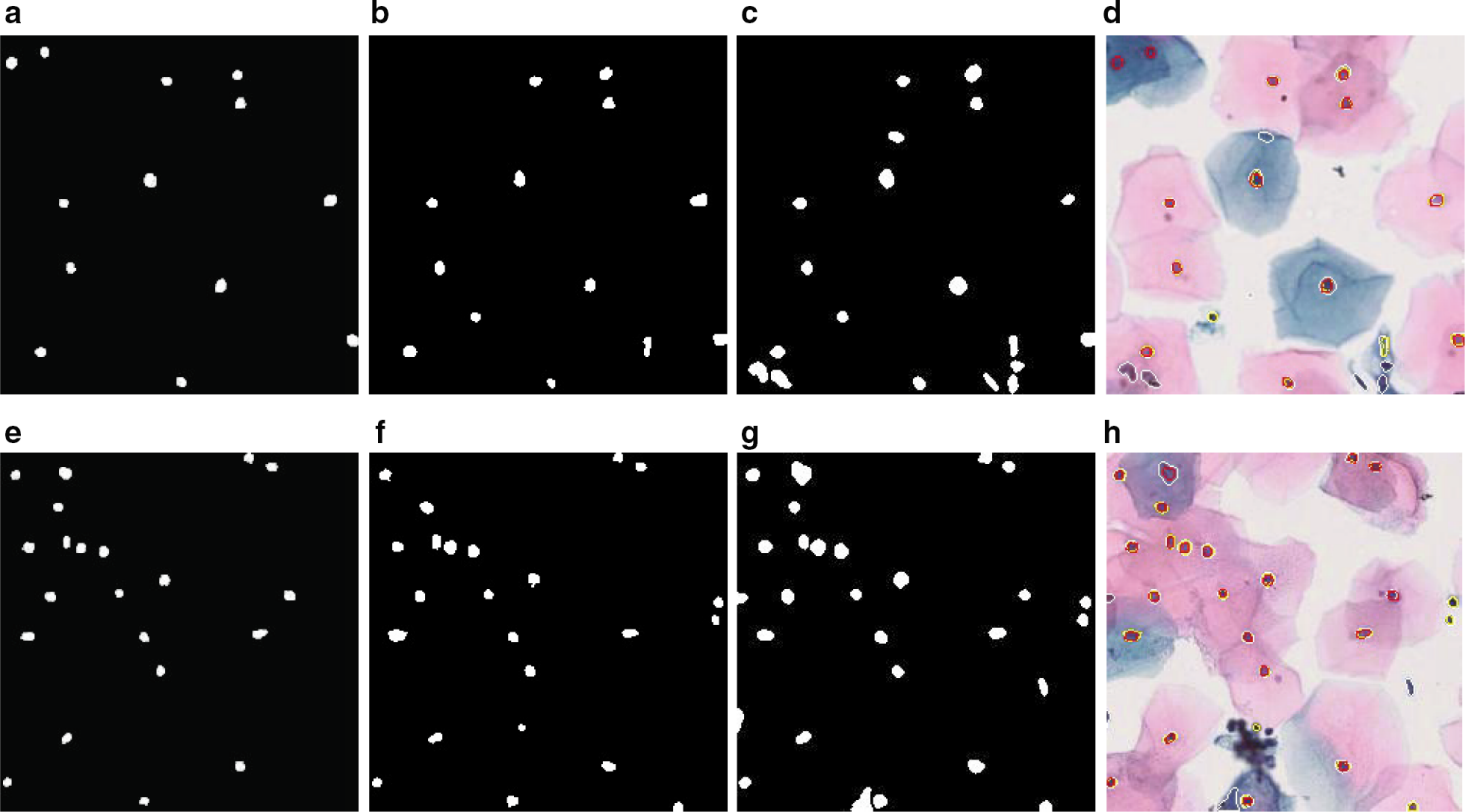
Qualitative comparison between our algorithm and Phoulady et al.’s algorithm. **d** & **h** directly compares the 3 kind of boundaries (Ours is yellow, Phoulady et al. [4] is white and Ground Truth is red)

While the approach of Phoulady et al. [4] can detect the presence of a nucleus properly, sometimes it fails to accurately delineate the boundary of the nucleus in pap smear images. This happens due to overzealous dilation at the very end of the algorithm, which unfortunately overextends their nuclei boundaries beyond the actual ones. Our proposed method is more conservative in this regard. Instead of general dilation, our nuclei recovery stage examines boundary pixels and extends the nuclei boundaries based on their average intensity, which is further scrutinized by a solidity check at the end. This results in delineating nuclei boundaries more accurately at the pixel level. When calculating the dice coefficient of each nucleus, before ultimately calculating the final object base measures, we noted down the values for each nucleus. We compared them with the values of the same nucleus from the output of the method of Phoulady et al. [4]. Our algorithm’s superior boundary delineation is evident from slightly better (2–3%) dice co-efficient values in most of the nuclei. Figure 3d and h show a qualitative comparison between the outputs of both methods on 2 separate images. Our nuclei boundaries are identified by yellow color, while white color indicates the boundaries of the method of Phoulady et al. [4] and red boundaries indicate the ground truth. We can see that, on most of the nuclei, the white boundary encircles the red one. On the other hand, the yellow boundaries are very close to the red boundaries, sometimes even getting overlapped by them.

### Our algorithm is more robust against outliers

Unlike the ISBI images, the real-life pap smear images contain many outliers (artifacts, superficial cells). These outliers often look very similar to actual nuclei, but they are, in fact, undesirables in the output image. Our algorithm is much more robust against these kinds of outliers than Phoulady et al.’s [4] algorithm. Their algorithm only uses minimum size and solidity to filter out noises and outliers, which fall short in filtering out all outliers, as is evident by their very low precision values. On the other hand, our algorithm has a separate noise removal stage followed by a more stringent check of contour properties (including minimum size, solidity, and inertia ratio). As a result, very few outliers are present in the final output images of our algorithm as compared to that of Phoulady et al.’s [4] algorithm. This stringent noise removal procedure ensures a very high precision value for our algorithm. Figure 3a–c & e–g compare the outputs from both methods to the respective ground truths. In the first image (top), our output contains only 2 outliers while the output of the algorithm of [4] has 8 (75% fewer outliers in ours). In the second image (bottom), our output contains 4 outliers while their output has 7 (43% fewer outliers in ours).

### Limitations

Our cervical nucleus segmentation approach has a slight drawback. There are seven tunable parameters of this method. In order to perform correct nuclei segmentation on any dataset, these parameters need to be tuned properly. Improper tuning of these parameters produces sub-par performance. Manually tuning these parameters is inefficient and makes our approach subjective to the dataset. To make this approach objective, we wrote a script for parameter tuning as described in section "Tunable parameters". The script itself can be found in our online repository [34]. This script requires a small set of annotated training images on which the script runs a grid search of different values of all the parameters to find the best combination thereof. The ability to adapt to different types of real cervical cytology datasets coupled with the flexibility to produce high precision, recall, F1-score, or AJI ultimately compensates for the drawback of requiring a small training dataset.

## Conclusion and future work

This paper introduces an algorithm to detect and segment nuclei from pap smear images based on local distinctive features. Here, each image was first adaptively thresholded and then passed through a convolution filter to filter out noises. Then contours from the images were filtered based on size, solidity, and inertial ratio, followed by an iterative method based on average contour intensity to recover the nucleus’s size and shape. The proposed algorithm produced satisfactory results, achieving the highest performance out of every work on the ISBI dataset while achieving reasonably well precision and F1-score on real pap smear images. For the pap smear test, recall is an important performance measure since higher recall means fewer missed nuclei. Thus our future work includes improving the recall while keeping the precision high on real cervical images and discovering an innovative method for cell clump and overlapping cytoplasm segmentation.

## Data Availability

All code and data can be found at the following link: https://github.com/caspianprince/CervicalNucl.

## Abbreviations

BSMMU: Bangabandhu Sheikh Mujib Medical University
CSF: Circular Shape Function
EM: Expectation Maximization
EDF: Extended Depth of Field
ISBI: International Symposium on Biomedical Imaging
MSER: Maximally Stable Extremal Regions
TP: True Positive
FP: False Positive
FN: False Negative
